# Impact of Arterial Calcification of the Lower Limbs on Long-Term Clinical Outcomes in Patients on Hemodialysis

**DOI:** 10.3390/jcm12041299

**Published:** 2023-02-06

**Authors:** Takayasu Ohtake, Ayaka Mitomo, Mizuki Yamano, Toshihiro Shimizu, Yasuhiro Mochida, Kunihiro Ishioka, Machiko Oka, Kyoko Maesato, Hidekazu Moriya, Sumi Hidaka, Milanga Mwanatambwe, Shuzo Kobayashi

**Affiliations:** 1 Department of Kidney and Transplant Center, Shonan Kamakura General Hospital, Kamakura 247-8533, Japan; 2 Regenerative Medicine, The Center for Cell Therapy & Regenerative Medicine, Shonan Kamakura General Hospital, 1370-1 Okamoto, Kamakura 247-8533, Japan; 3 Shonan Research Institute of Innovative Medicine (sRIIM), Kamakura 247-8533, Japan; 4 Department of Nephrology, Tokyo Nishi Tokushukai Hospital, Tokyo 196-0003, Japan; 5 Department of Pathology, University of Mbuji Mayi, Mbujimayi 433, Congo; 6 International Division of Tokushukai of Medical Corporation, Tokushukai, Tokyo 188-0013, Japan

**Keywords:** lower extremity artery disease, hemodialysis, limb amputation, cardiovascular event, arterial calcification

## Abstract

Lower limbs’ arterial calcification is significantly associated with the clinical severity of lower extremity artery disease (LEAD) in patients undergoing hemodialysis (HD). However, the association between arterial calcification of the lower limbs and long-term clinical outcomes in patients on HD has not been elucidated. Calcification scores of the superficial femoral artery (SFACS) and below-knee arteries (BKACS) were quantitatively evaluated in 97 HD patients who were followed for 10 years. Clinical outcomes, including all-cause and cardiovascular mortality, cardiovascular events, and limb amputation were evaluated. Risk factors for clinical outcomes were evaluated using univariate and multivariate Cox proportional hazard analyses. Furthermore, SFACS and BKACS were divided into three groups (low, middle, and high), and their associations with clinical outcomes were evaluated using Kaplan–Meier analysis. SFACS, BKACS, C-reactive protein, serum albumin, age, diabetes, presence of ischemic heart disease, and critical limb-threatening ischemia were significantly associated with 3-year and 10-year clinical outcomes in the univariate analysis. Multivariate analysis showed that SFACS was an independent factor associated with 10-year cardiovascular events and limb amputations. Kaplan–Meier life table analysis showed that higher SFACS and BKACS levels were significantly associated with cardiovascular events and mortality. In conclusion, long-term clinical outcomes and the risk factors in patients undergoing HD were evaluated. Arterial calcification of the lower limbs was strongly associated with 10-year cardiovascular events and mortality in patients undergoing HD.

## 1. Introduction

Accelerated atherosclerosis is one of the most important complications and plays a central role in the pathogenesis of cardiovascular disease in patients undergoing hemodialysis (HD). Lindner et al. described accelerated atherosclerosis in HD patients approximately 50 years ago [1]. Since then, much evidence concerning atherosclerotic cardiovascular complications in HD patients has been provided. Among atherosclerotic comorbidities, lower extremity artery disease (LEAD) significantly impacts mortality in HD patients [2]. In a previous study, the 1-year survival rate fell to almost 50% after a major amputation in the lower limb due to LEAD [3], which was comparable to the 1-year survival rate after acute myocardial infarction (AMI) in HD patients [4].

Atherosclerosis in patients with chronic kidney disease (CKD) progresses with composite risk factors including traditional cardiovascular risk factors such as hypertension, dyslipidemia, aging, diabetes mellitus, and characteristic risk factors in CKD including oxidative stress, insulin resistance, uremic toxins, calcium–phosphate metabolism abnormality, and microinflammation [5]. An active atherosclerotic process begins in the early stages of CKD, and atherosclerotic organ damages deteriorate along with the decreasing renal function. Decreased glomerular filtration ratio (GFR) is an independent risk factor for lacunar infarction [6]. By the time of initiation of renal replacement therapy, approximately 50% of patients with CKD have coronary artery stenosis, regardless of the presence or absence of symptoms and/or signs [7]. One characteristic pathophysiologic feature of atherosclerosis in CKD patients is combined injuries of intima and media in vessels, i.e., endothelial dysfunction and medial calcification. This constructs “vascular failure”, leading to several organ damages in the advanced stages of CKD. Another characteristic feature of atherosclerosis in CKD patients is “polyvascular disease”. Polyvascular disease is defined as a coexistent symptomatic arterial disease in two or three territories (coronary, cerebral, and/or peripheral arteries). As shown in our previous studies [8], many patients undergoing HD have polyvascular disease.

Historically known as Mönkeberg calcification [9], vascular calcification is a characteristic feature of atherosclerosis in HD patients. Arterial calcification in the peripheral arteries is widely distributed in all portions of the body in HD patients, and aortic and cardiac valve calcifications are also observed in long-standing HD patients. Vascular calcification progresses along with the duration of HD, and microinflammation and an abnormal calcium phosphate metabolism are significant predictors of the progression of vascular calcification in patients undergoing HD [10].

Vascular calcification significantly affects future cardiovascular events and mortality in HD patients. Coronary artery calcification has been extensively studied and is significantly associated with future cardiovascular events and/or mortality in HD patients [10,11,12,13,14,15,16]. Aortic calcification was also reported to be significantly associated with future cardiovascular events and/or mortality [17,18,19,20,21,22]. Therefore, vascular calcification has a deleterious effect on the prognosis of patients on HD. 

Although coronary artery and aortic calcifications have been extensively evaluated in HD patients, arterial calcification of the lower limbs, an important characteristic of LEAD in HD patients, has not been evaluated. Previously, we quantitatively evaluated lower limbs’ arterial calcification using multi-detector computed tomography (MDCT) and reported that arterial calcification of the lower limbs was closely associated with the prevalence and severity of LEAD in HD patients [8]. However, the association between arterial calcification of the lower limbs and clinical outcomes in patients on HD has not been elucidated. Therefore, we evaluated the association between arterial calcification of the lower limbs and long-term clinical outcomes, including all-cause and cardiovascular mortality, cardiovascular events, and limb amputation in HD patients. We are the first to provide evidence that arterial calcification of the lower limbs significantly impacts long-term clinical outcomes in HD patients.

## 2. Materials and Methods

### 2.1. Patients Enrollment and Purpose of This Study

Ninety-seven HD patients who underwent non-enhanced 64-row MDCT to evaluate the calcification score (CS) of the lower limb arteries were registered in 2008, as described in our previous study [8]. Patients were followed up for 10 years, and outcomes, including all-cause mortality, cardiovascular mortality, cardiovascular events, and limb amputation, were evaluated. 

This study aimed to elucidate (1) the long-term clinical outcomes for up to 10 years, (2) the risk factors for long-term clinical outcomes, and (3) the impact of arterial calcification of the lower limbs on clinical outcomes in HD patients.

This study was conducted in accordance with the Declaration of Helsinki. Data were collected from electronic records, and this study was conducted after approval from the Institutional Review Board (IRB) (approval number: TGE01879-024); written informed consent was obtained from the patients included in the previous study [8]. In this follow-up study, the IRB permitted an opt-out approach. The patients could opt out after the research project was made available on our hospital website.

### 2.2. Lower Limbs’ Arterial Calcification

Patients underwent non-contrast-enhanced MDCT scanning of the arteries of the lower limbs using a 64-row MDCT scanner (SOMATOM sensation cardiac 64, SIEMENS, Germany), as previously mentioned [8]. The scoring of calcification for bilateral superficial femoral arteries (SFA) and below-knee arteries (BKA), including the anterior tibial, posterior tibial, and peroneal arteries, was performed using standardized calcium scoring software (Aquarius Net Station, TERARECON) by investigators who were blinded to the results of the patients’ clinical assessment and Fontaine’s severity classification. The CS of SFA (SFACS) and BKA (BKACS) was determined and expressed as the Agatston score, according to the method described by Agatston et al. [23].

### 2.3. Measurement of Ankle–Brachial Pressure Index and Toe–Brachial Pressure Index

To evaluate functional blood flow in the lower extremities, we conducted physiological measurements, including ankle–brachial pressure index (ABI) and toe–brachial pressure index (TBI). ABI and TBI were measured, as previously reported, using the ABI form (Colin, Co., Ltd., Komaki, Japan) [8]. Normal ABI and TBI were reported to be 0.9–1.3 and 0.6<, respectively, in the general population [24]. However, in our previous study, we confirmed that the best ABI cut-off value for detecting LEAD in HD patients was 1.06 [8]. Sensitivity and specificity of ABI values < 1.06 for detecting LEAD in dialysis patients were 80.0% and 98%, respectively [8]. Therefore, an ABI cut-off value of 1.06 was used in this study. The cut-off value for TBI was <0.6, as used in our previous study.

### 2.4. Patient Assessment and Group Assignment

Information about symptoms of LEAD, including chills, numbness, ischemic claudication, and resting pain, was collected through patient interviews. The previous history of intervention (percutaneous peripheral intervention or bypass surgery) or amputation for LEAD was also recorded. All patients underwent physiological examination for skin color; warmth; pulse examination of the femoral, popliteal, dorsal, and posterior tibial arteries; and skin lesions, including ulcers and gangrene. Symptomatic information, previous history of intervention or amputation, and physiological examinations, including ABI and TBI, were used to assign limb ischemia according to the LEAD severity classification criteria set by Fontaine et al. [25]. Patients with ischemic symptoms with at least one abnormality among ABI < 1.06 and TBI < 0.6 or who had an apparent previous history of intervention or amputation of lower limbs were defined as having LEAD. In cases with clinical symptoms, including chillness or numbness without ABI < 1.06 or TBI < 0.6, skin perfusion pressure (SPP) was measured using laser Doppler (PAD 3000: Kaneka, Tokyo, Japan). The PAD 3000 automatically measures the SPP using the laser Doppler transmitter and detector, which were set with a pressure cuff. Two points were measured in each patient: (a) a point between the first and second metatarsal bones in the instep and (b) a front middle point in the sole. After inflating the cuff pressure to stop skin perfusion, the cuff pressure was deflated, and the point when skin perfusion restarted was measured. SPP was expressed as the pressure required to restart skin perfusion. If the SPP value was less than 50 mmHg, the patient was considered Fontaine category I. Three doctors confirmed the Fontaine category classification using the data files on ABI, TBI, and SPP findings and their clinical symptoms. 

### 2.5. Clinical and Laboratory Parameters

Clinical information was collected from medical records regarding age, sex, dialysis duration, cause of renal failure, dyslipidemia, hypertension, diabetes mellitus (DM), and comorbid diseases, including ischemic heart disease (IHD) and stroke. Laboratory parameters, including serum albumin, calcium, inorganic phosphate, C-reactive protein (CRP), total cholesterol, triglyceride, high-density lipoprotein cholesterol, low-density lipoprotein cholesterol, and plasma fibrinogen, were measured using blood samples at the start of the first HD of the week within 2 weeks of MDCT examination. Serum calcium levels were recorded after correcting for albumin. Medications, including antiplatelet drugs, angiotensin receptor blockers, angiotensin-converting enzyme inhibitors, statins, phosphate binders, and vitamin D, were also registered.

### 2.6. Treatment Strategy for LEAD

LEAD was treated according to the Trans-Atlantic Inter-Society Consensus Document on Management of Peripheral Arterial Disease guidelines [26]. Patients with LEAD were treated with either medications, including antiplatelet drugs, or prostaglandin I_2_ analogues or both. In patients with intermittent claudication, resting pain, and/or ulceration or gangrene (Fontaine II–IV), the culprit arterial stenotic/obstructive lesions were evaluated using a combination of ultrasonography, contrast-enhanced MDCT, non-contrast-enhanced magnetic resonance imaging, or angiography. Re-vascularization treatment (endovascular therapy or bypass surgery) was performed for Fontaine category II patients if the patient had severe claudication, and the re-vascularization procedure was considered mandatory. 

In patients with critical limb-threatening ischemia (CLTI), re-vascularization was essentially performed to improve the blood flow in the ischemic limb. If blood flow improved to ≥40 mmHg at the SPP level, surgical debridement therapy and/or vacuum-assisted closure (VAC) were performed. Antibiotic therapy was also administered if the patients had a local infection that was confirmed by a bacterial culture test.

Limb amputation was considered in cases of intractable pain or severe infection with rapid worsening and progression to a septic state. Amputation was also considered when the patient had no optional intractable wound, and rehabilitation after limb amputation was considered mandatory for early back to social life.

### 2.7. Patient Outcomes

Clinical outcomes, including all-cause mortality, cardiovascular mortality, cardiovascular events, and limb amputation, were obtained from electronic records: these outcomes were evaluated 3 and 10 years after registration. Limb amputation was registered as major or minor amputation. Major and minor amputation were defined depending on the amputation site, above or below the ankle joint, respectively. Major amputation was further divided into above-knee (AK) and below-knee (BK) amputation. All deaths during the observation period were recorded with the cause of death. 

All-cause mortalities included cardiovascular deaths, deaths due to infectious diseases, and deaths due to other causes such as malignancy. Cardiovascular deaths included IHD, heart failure, sudden death, stroke, and fatal arrhythmia. Cardiovascular events included new-onset heart failure, AMI, sudden death, stroke, and arrhythmia. New-onset arrhythmia as a cardiovascular event included atrial fibrillation, ventricular tachycardia, sick sinus syndrome, grade 2 or 3 atrioventricular block, and supraventricular or ventricular extra-systole.

The association between the severity of arterial calcification in the lower limbs and clinical outcomes was evaluated using stratified SFACS and BKACS. The SFACS and BKACS were divided into three categories: i.e., low, middle, and high. SFACS was divided into low, <300 (n = 31); middle, ≥300 to <3000 (n = 34); and high, ≥3000 (n = 32). BKACS was divided into low, <100 (n = 25); middle, ≥100 to <1000 (n = 35); and high, ≥1000 (n = 37). 

### 2.8. Statistical Analysis

All data were presented as mean ± standard deviation (SD). Data on each patient’s dominant side of the leg for LEAD were selected and used for ABI, TBI, SFACS, and BKACS analyses. Patients were divided into LEAD (-) patients or LEAD (+) patients, and LEAD (+) patients were further divided according to the Fontaine classification. Group mean values were compared using a two-tailed unpaired Student’s *t*-test or Mann–Whitney U-test. Skewed variables or variables with a wide SD, including CRP, SFACS, and BKACS, were log-transformed before statistical analysis. Categorical variables were compared using the chi-square and Fisher’s tests. Univariate Cox proportional hazard analysis was performed to identify the significant risk factors for clinical outcomes. Multivariate Cox proportional hazard analysis was performed to identify independent risk factors for the clinical outcomes. Since we had confirmed a strong positive correlation between SFACS and BKACS (r = 0.797, *p* < 0.0001) in our previous study [8], multivariate analysis was performed in 2 patterns. Model 1 included SFACS and significant variables in univariate analysis, and model 2 included BKACS and significant analysis in univariate analysis, respectively. The influence of the severity of arterial calcification of the lower limbs on patient outcomes, including all-cause mortality, cardiovascular mortality, cardiovascular events, and limb amputation, was assessed using the Kaplan–Meier life table analysis and log-rank test. SPSS version 11.0 software (SPSS Inc., Chicago, IL, USA) was used for data analysis on a personal computer, and *p* < 0.05 was considered significant.

## 3. Results

### 3.1. Patient Characteristics and Fontaine Group Assignment

Approximately, half of the enrolled patients (50/97 patients, 51.5%) had LEAD, and there were 31, 8, and 11 patients in Fontaine categories I, II, and IV, respectively (Table 1) Atherosclerotic comorbidities, including IHD and stroke, were significantly higher in patients with LEAD, irrespective of the Fontaine category. SFACS and BKACS were significantly higher in patients with LEAD than in those without LEAD, even in the early stage of LEAD (Table 1) SFACS and BKACS significantly increased along with the progression of the clinical severity of LEAD (Fontaine category I vs. Fontaine category IV, *p* < 0.01). TBI in LEAD patients was significantly lower than in non-LEAD patients and significantly decreased with the progression of limb ischemia. Similarly, ABI values in patients with LEAD were significantly lower than those in patients without LEAD. However, ABI did not show a concomitant decrease with the progression of limb ischemia. Antiplatelet drugs were administered to all patients with CLTI, whereas 51.6% of Fontaine I patients received antiplatelet therapy.

### 3.2. Treatment of LEAD

Endovascular treatment (EVT) was performed in all Fontaine IV patients with CLTI (n = 11). The frequency of EVT for AK and BK arteries was almost the same. Distal bypass surgery (distal anastomosis in BK arteries) was performed in three CLTI patients with BK arterial occlusion in whom EVT for BK arteries was unsuccessful.

### 3.3. Patient Outcome

Worsening of limb ischemia from non-CLTI to CLTI during a 3-year follow-up period was seen in one (2.1%) patient without LEAD and three (9.7%) Fontaine category Ⅰ patients. Symptoms of intermittent claudication were not observed in these patients before worsening to CLTI.

Twenty-seven (27.8%) and fifty-eight (60.0%) patients died at the end of the 3-year and 10-year observations, respectively [Table 2]. By the end of the 3-year follow-up period, 9 (19.1%) out of 47 patients without LEAD, 8 (25.8%) out of 31 Fontaine category I patients, 2 (25.0%) out of 8 Fontaine category II patients, and 8 (72.7%) out of 11 Fontaine category IV patients died. The mortality rate was significantly higher in LEAD patients, irrespective of the Fontaine category, than in non-LEAD patients during the 3-year follow-up period. A remarkably high mortality rate was observed in Fontaine category IV patients (72.7%) [Table 2]. 

Among 27 patients who died within 3 years, 14 (51.9%), 4 (14.8%), and 9 (33.3%) patients died of cardiovascular causes, infectious diseases, and other causes, respectively. In 10-year all-cause deaths, the mortality rates between non-LEAD patients and patients with non-CLTI LEAD (Fontaine category I) were not statistically different. However, the mortality rate in Fontaine category IV patients was significantly higher than those in patients without LEAD and patients with early-stage LEAD. Cardiovascular deaths were the most dominant cause of death in Fontaine category IV patients in the 3-year and 10-year observation periods [Table 2].

Regarding cardiovascular events, CLTI was significantly associated with high cardiovascular events at the end of the 3-year and 10-year observations. Heart failure and arrhythmia were significantly more common in patients with LEAD than in those without LEAD during the 3-year observation period. As for limb amputation, nine limb amputations occurred during the 3-year follow-up period. The amputation rate was significantly higher in patients with LEAD than in non-LEAD patients; similarly, the amputation rate was significantly higher in Fontaine category II and IV patients than in category I patients. Four amputations were performed on Fontaine category I patients at the end of the 3-year observation period. Three patients showed sudden progression to CLTI during the 3-year follow-up period. One patient experienced an AK amputation in one leg and a minor amputation in the contralateral leg. 

### 3.4. Risk Factors for 3-Year and 10-Year Clinical Outcomes

Table 3 and Table 4 show the significant risk factors for patient outcomes at 3 and 10 years, respectively. Among several variables, CRP, serum albumin, age, and a previous history of IHD, SFACS, BKACS, and CLTI were significantly associated with patient outcomes in a univariate Cox proportional hazard analysis. The univariate Cox proportional hazard analysis showed that CRP and CLTI were significant risk factors for all clinical outcomes, including all-cause mortality, cardiovascular mortality, cardiovascular events, and limb amputation. SFACS was a significant risk factor for cardiovascular events during the 3-year observation period and all-cause mortality, cardiovascular mortality, cardiovascular events, and limb amputation during the 10-year observation period. BKACS was a significant risk factor for all clinical outcomes at the 3-year and 10-year observations in the univariate analysis. Multivariate Cox proportional hazard analysis revealed independent risk factors for clinical outcomes at the end of the 3-year and 10-year observations. For the 10-year observation, SFACS was an independent risk factor for cardiovascular events and limb amputation, independent of CLTI. 

### 3.5. Impact of SFACS and BKACS on 10-Year Clinical Outcomes

**Figure 1:**
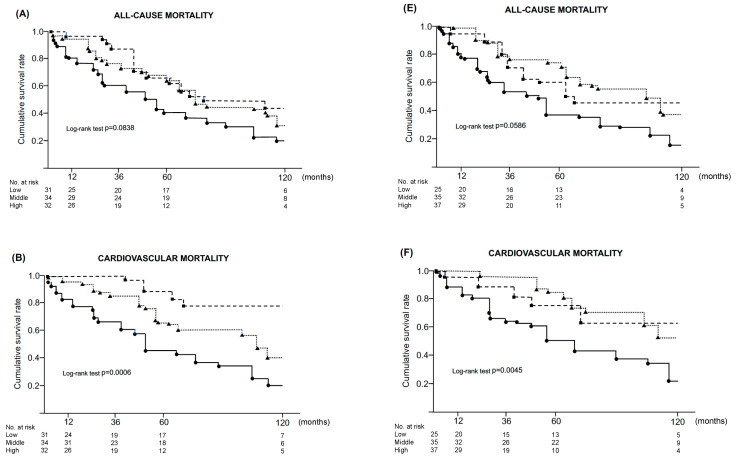

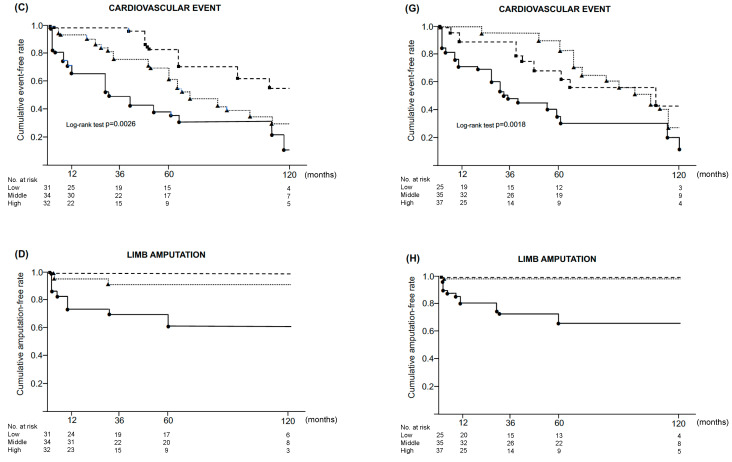
Stratified calcification scores and clinical outcomes. SFACS and BKACS were divided into 3 groups according to calcification scores, i.e., low (▪), middle (▲), and high (●). (**A**,**E**) all-cause mortality, (**B**,**F**) cardiovascular mortality, (**C**,**G**) cardiovascular events, and (**D**,**H**) limb amputation. (**A**–**D**) SFACS and (**E**–**H**) BKACS. Abbreviations: SFACS, superficial femoral artery calcification score; BKACS, below-knee arteries calcification score.

Stratified SFACS and BKACS significantly impacted cardiovascular mortality and cardiovascular events, respectively (Figure 1). The higher SFACS and BKACS groups showed significantly higher rates of cardiovascular mortality and events, respectively ((B), (C), (F), (G), *p* < 0.01). Stratified SFACS and BKACS showed borderline associations with all-cause mortality (although these were not statistically significant). Regarding limb amputation, the high SFACS and BKACS groups showed worse survival curves than the low SFACS and BKACS groups. However, because amputation was not performed in some groups (the low SFACS group and the low and middle BKACS groups) during the observation period, statistically significant differences were not observed in the life table analysis.

## 4. Discussion

Our study provided the first evidence that quantitatively evaluated arterial calcification of the lower limbs was significantly associated with 10-year cardiovascular events and mortality in patients undergoing HD. The risk factors for middle, and long-term clinical outcomes including all-cause mortality, cardiovascular mortality, cardiovascular events, and limb amputation were CRP, serum albumin, age, DM, presence of IHD, CLTI, and vascular calcification score, including SFACS and BKACS, obtained by a univariate Cox proportional hazard analysis. A multivariate Cox proportional hazard analysis showed that the CRP level was a strong and independent predictor of all-cause mortality, cardiovascular mortality, and cardiovascular events in a 10-year follow-up study. Vascular calcification, especially SFACS, was an independent predictor of cardiovascular events and limb amputation in the 10-year follow-up in multivariate analysis.

Only a few reports have quantitatively evaluated arterial calcification of the lower limbs and its association with clinical outcomes in CKD patients. Mizuiri et al. quantitatively evaluated the calcification score of common iliac artery (CIA) and coronary artery using MDCT in 145 non-dialysis CKD patients [27]. They evaluated the association between the vascular calcification of two different sites with the progression to renal replacement therapy. Coronary artery calcification significantly predicted the progression to renal replacement therapy. However, CIA calcification did not predict the renal prognosis. Although they quantitatively evaluated CIA calcification, they did not evaluate the association between CIA calcification and clinical outcomes, including all-cause and cardiovascular mortality, cardiovascular events, and limb amputation, as evaluated in our study. Several atherosclerotic risk factors are known to be associated with different distribution pattern in peripheral artery disease. Smoking is associated with more proximal atherosclerotic distribution (more elastic elements such as the iliac artery), whereas kidney dysfunction shows a more centrifugal lesion pattern (more muscular elements such as SFA and BKA) [28]. Distal muscular arteries may be more strongly affected in uremia by vascular calcification via the osteogenic differentiation of vascular smooth muscle cells (VSMCs). Therefore, vascular calcification in more distal arterial portion of lower limbs may better predict the clinical outcomes in patients undergoing HD as shown in our study.

HD patients with LEAD have a very high rate of atherosclerotic comorbidity, including IHD and stroke. Furthermore, more than half of these deaths were of cardiovascular origin. Therefore, adequate treatment of the target organ damage due to atherosclerosis (not only LEAD but also the cardiovascular and cerebrovascular systems) may be very important.

Drug administration was insufficient in HD patients with early-stage LEAD. This may be due to the nature of LEAD, that is, asymptomatic or very mild symptoms in its early stage, and inappropriate judgment of screening tests such as ABI (influenced by vascular calcification). As we previously provided, the cut-off value of ABI to detect LEAD may be set at 1.06 for HD patients. Vascular calcification may lead to a misunderstanding of ABI values, and many HD patients may be misdiagnosed as not having LEAD by applying normal ABI values (0.9–1.3) in the general population. Since advanced stages of LEAD result in deleterious outcomes in patients undergoing HD, early diagnosis is extremely important. Furthermore, some patients showed sudden worsening from non-CLTI to CLTI without showing gradual worsening. Therefore, although clarifying whether early drug intervention in the early stage of LEAD could prevent the progression of LEAD would be relevant, early diagnosis of LEAD may be the most important thing in HD patients.

The progression of vascular calcification via an inflammatory mechanism with a background of severe calcium–phosphate abnormalities plays an important role in accelerated atherosclerosis in patients undergoing HD. Vascular calcification and inflammation are thought to be associated. Increased uptake of phosphate by VSMCs via the Pit-1 co-transporter under elevated phosphate induces Runx2 upregulation and promotes the osteogenic differentiation and calcification of VSMCs. The inflammatory cytokine, tumor necrosis factor-alfa, elevates intracellular cyclic adenosine monophosphate, upregulates Pit-1 messenger RNA, and promotes the osteogenic differentiation of VSMCs [29]. In accordance with the primary role of microinflammation at the cellular level, microinflammation, represented by CRP, is a clinically important factor associated with vascular calcification. The CRP level was an independent factor associated with SFACS in our previous study [8]. Furthermore, CRP is strongly associated with the progression of calcium deposition in vascular cells [10]. In the present study, CRP further demonstrated its role as a strong independent predictor of future clinical outcomes, including all-cause mortality, cardiovascular mortality, and cardiovascular events.

Stratified SFACS and BKACS indicated that the higher calcification group significantly affected long-term clinical outcomes, including cardiovascular events and mortality. The higher calcification group for both SFACS and BKACS also showed worse clinical outcomes in limb amputation. Therefore, delaying or improving vascular calcification is thought to be very important for improving cardiovascular outcomes in HD patients. Several trials have been performed to delay the progression of vascular calcification using non-calcium-containing phosphate binders [30,31,32,33,34,35], optimal and strict phosphate control using non-calcium-based phosphate binders [36], low-dose active vitamin D plus cinacalcet [37], and modification of dialysate calcium concentration [38]. These trials, including our previous study [30], succeeded in delaying the progression of vascular calcification. However, delaying or improving vascular calcification has not been clearly proven to result in improved cardiovascular events and/or mortality rates in prospective interventional randomized controlled trials in dialysis patients [39,40]. In consideration of atherosclerosis as an inflammatory process, not only delaying the progression of vascular calcification but also optimally controlling the underlying pathophysiology in HD patients, such as microinflammation, may be necessary to improve the prognosis of HD patients.

As for endovascular treatment, recent advances enabled drug-eluting balloon (DEB) angioplasty for LEAD. In cases of coronary artery stenosis as well, efficacy of DEB angioplasty compared with uncoated balloon angioplasty was reported in the treatment of critical arterial stenosis and/or occlusions in lower limbs [41,42,43]. The rates of primary vessel patency, late lumen loss, and target lesion revascularization were superior in DEB angioplasty compared with uncoated balloon angioplasty, respectively [41,42]. Heideman et al. reported that long-term all-cause mortality, rates of amputation or death, and cardiovascular events or death were significantly reduced after the use of paclitaxel coated devises compared with uncoated devices for the treatment of CLTI [43]. Teymen et al. reported the efficacy of DEB angioplasty in patients with end stage renal disease [44]. However, their study was a retrospective, single-center study. Therefore, a convincing result for the efficacy of DEB angioplasty has not been obtained in CKD patients. A future, randomized, controlled interventional trial for the treatment of CLTI in HD patients with highly calcified vessels is needed to clarify the issue. DEB became commercially available in the autumn of 2018 in Japan (at the end of the follow-up in our study). Therefore, we could not evaluate the efficacy of DEB angioplasty in HD patients.

In conclusion, 60% of patients on maintenance HD died during the 10-year follow-up period. The risk factors for long-term clinical outcomes were CRP, serum albumin, age, DM, presence of IHD, CLTI, and vascular calcification score, including SFACS and BKACS, by a univariate Cox proportional hazard analysis. The CRP level was a strong and independent predictor of all-cause mortality, cardiovascular mortality, and cardiovascular events in a 10-year follow-up study. Arterial calcification of the lower limbs significantly predicted 10-year cardiovascular events and mortality (but not all-cause mortality) in HD patients. Although the number of patients was rather small, and this was a single-center observational study, our study provided new insights in the field of vascular medicine. Quantitative evaluation of arterial calcification of the lower limbs using MDCT for all HD patients may not be practical. However, to understand and keep in mind the impact of arterial calcification of the lower limbs on long-term clinical outcomes may contribute to improving the quality of care provided for HD patients with LEAD. Future prospective interventional trials may be needed, regarding whether delaying or improving arterial calcification of the lower limbs may improve long-term clinical outcomes in patients undergoing HD.

## Figures and Tables

**Table 1:** Baseline characteristics of the patients.

	LEAD (-)	LEAD (+) Fontaine Category		
	(n = 47)	I (n = 31)	II (n = 8)	IV (n = 11)
Age (yr)	63.0 ± 13.4	72.5 ± 9.0 **	71.4 ± 6.6	73.1 ± 10.5 *
Male/female (n)	31:16	17:14	8:0	7:4
HD duration (months)	53.6 ± 51.3	74.5 ± 70.1	171.1 ± 144.7	74.9 ± 70.8
Risk factors n, (%)				
Diabetes	13 (27.7)	12 (38.7)	4 (50.0)	9 (81.8) **
Hypertension	44 (93.6)	28 (90.3)	8 (100)	11 (100)
Dyslipidemia	5 (10.6)	8 (25.8)	0 (0)	5 (45.5) *
Smoking	8 (17.0)	4 (12.9)	1 (12.5)	1 (9.0)
Comorbidity n, (%)				
IHD	3 (6.4)	17 (54.8) **	4 (50.0) *	6 (54.5) **
Stroke	1 (2.1)	7 (22.6) *	3 (37.5) *	4 (36.3) *
Blood pressure (mmHg)				
Systolic	138.1 ± 18.3	143.0 ± 21.4	128.2 ± 198.9	136.7 ± 20.6
Diastolic	79.5 ± 12.3	78.3 ± 12.5	68.9 ± 8.8	72.6 ± 20.6
Laboratory valuables				
CRP (mg/dL)	0.18 ± 0.31	0.42 ± 0.53	0.70 ± 1.43 *	6.91 ± 16.5 **
Albumin (g/dL)	3.7 ± 0.5	3.7 ± 0.5	3.6 ± 0.2	3.3 ± 0.4 *
Fibrinogen (mg/dL)	319.1 ± 88.2	329.8 ± 91.6	312.2 ± 84.8	424.5 ± 91.5 **
Ca (mg/dL)	9.0 ± 0.8	9.3 ± 0.7	9.2 ± 1.1	9.2 ± 0.9
Pi (mg/dL)	5.7 ± 1.5	5.4 ± 1.4	4.9 ± 1.2	6.0 ± 2.0
T.Chol (mg/dL)	166.6 ± 44.8	162.9 ± 41.7	136.8 ± 20.1	144.0 ± 35.1
TG (mg/dL)	95.4 ± 44.8	106.4 ± 86.1	104.1 ± 41.3	89.8 ± 27.6
HDL-C (mg/dL)	50.1 ± 17.9	48.3 ± 13.3	39.4 ± 11.9	44.8 ± 12.8
LDL-C (mg/dL)	84.7 ± 33.3	79.0 ± 28.6	65.9 ± 17.7	74.4 ± 32.3
ABI	1.21 ± 0.09	0.89 ± 0.31 **	0.82 ± 0.27	0.82 ± 0.26
TBI	0.82 ± 0.19	0.44 ± 0.22 **	0.45 ± 0.25	0.16 ± 0.13 *^#^
Calcification score				
SFACS	865.1 ± 2279.6	4307.3 ± 4471.5 ***	4680.3 ± 4682.7 ***	9579.7 ± 5856.9 ***^#^
BKACS	619.9 ± 1437.1	2035.9 ± 2993.7 ***	7151.1 ± 7438.7 ***	7565.9 ± 5808.0 ***^#^
Medication				
Antiplatelet drugs, n (%)	3 (6.4)	16 (51.6) **	8 (100) **	11 (100) **
Prostaglandin I2 analogue	0 (0)	6 (19.5) **	1 (12.5)	5 (54.5) **
ARB	39 (83.0)	15 (48.4)	5 (62.5)	4 (36.4) *
ACE-I	3 (6.4)	2 (6.5)	0 (0)	0 (0)
Statin	5 (10.6)	8 (3.2)	0 (0)	5 (45.5) *
Phosphate binder	0 (0)	6 (19.4)	1 (12.5)	5 (54.5)
Calcium carbonate	33 (70.2)	18 (58.1)	5 (62.5)	6 (54.5)
Sevelamer	10 (21.3)	8 (25.8)	6 (75.0) **	3 (27.3)
Vitamin D	24 (51.0)	18 (58.1)	5 (62.5)	7 (63.6)

* *p* < 0.05, ** *p* < 0.01, *** *p* < 0.001 vs. LEAD (-), # *p* < 0.01 vs. Fontaine category 1. Abbreviations: LEAD, lower extremity artery disease; HD, hemodialysis; IHD, ischemic heart disease; CRP, C-reactive protein; Ca, calcium; Pi, inorganic phosphate; T.Chol, total cholesterol; TG, triglyceride; HDL-C, high-density lipoprotein; LDL-C, low-density lipoprotein; ABI, ankle–brachial index; TBI, toe–brachial index; SFACS, superficial femoral artery calcification score; BKACS, below-knee artery calcification score; ARB, angiotensin receptor blocker; ACE-I, angiotensin converting enzyme inhibitor.

**Table 2:** Three-year and ten-year clinical outcomes.

	LEAD (-)	LEAD (+) Fontaine Category		
	(n = 47)	I (n = 31)	II (n = 8)	IV (n = 11)
3-year death, n, (%)	9 (19.1)	8 (25.8) **	2 (25.0) **^##^	8 (72.7) **^##^
Cardiovascular	4 (8.5)	4 (12.8)	0 (0)	6 (54.5) ***^##^
Infection	0 (0)	2 (6.5)	1 (12.5)	1 (9.1)
Other	5 (10.6)	2 (6.5)	1 (12.5)	1 (9.1)
10-year death, n, (%)	24 (51.1)	21 (67.7)	4 (50.0)	9 (81.8) **^#^
Cardiovascular	15 (31.9)	11 (35.4)	3 (37.5)	8 (72.7) *^#^
Infection	4 (8.5)	6 (19.4)	0 (0)	0 (0)
Other	5 (10.6)	4 (12.9)	1 (12.5)	1 (9.1)
3-year cardiovascular event, n, (%)	8 (17.2)	6 (19.4)	2 (25.0)	9 (81.9) ***^##^
Heart failure				
AMI	0 (0)	0 (0)	0 (0)	1 (9.1)
Sudden death	2 (4.3)	1 (3.2)	0 (0)	1 (9.1)
Stroke	2 (4.3)	1 (3.2)	0 (0)	1 (9.1)
Arrythmia	2 (4.3)	2 (6.5)	2 (25.0)	3 (27.3) *
10-year cardiovascular event, n, (%)	24 (50.9)	14 (45.3)	8 (100) *	10(90.9) *^##^
Heart failure	5 (10.6)	3 (9.7)	0 (0)	3 (27.2)
AMI	0 (0)	2 (6.5)	1 (12.5)	2 (18.2)
Sudden death	5 (10.6)	2 (6.5)	1 (12.5)	1 (9.1)
Stroke	5 (10.6)	2 (6.5)	2 (25.0)	2 (18.2)
Arrythmia	9 (19.1)	5 (16.1)	4 (50.0)	2 (18.2)
3-year amputation, n, (%)	0 (0)	4 (12.9) **	1 (12.5) **^##^	4 (36.4) **^##^
Major	0 (0)	2 (6.4)	0 (0)	2 (18.2)
Minor	0 (0)	2 (6.5)	1 (12.5)	2 (18.2)
10-year amputation, n, (%)	1 (2.1)	4 (12.9) **	1 (12.5) **^##^	7 (63.6) **^##^
Major	0 (0)	2 (6.4)	0 (0)	4 (36.4)
Minor	1 (2.1)	2 (6.5)	1 (12.5)	3 (27.2)

* *p* < 0.05, ** *p* < 0.01, *** *p* < 0.001 vs. LEAD (-), # *p* < 0.05, ## *p* < 0.01 vs. Fontaine category 1. Abbreviations: LEAD, lower extremity artery disease; AMI, acute myocardial infarction.

**Table 3:** Cox proportional hazard analysis for association with 3-year clinical outcomes.

	Univariate			Multivariate					
					Model 1			Model 2	
	HR	95% CI	*p*	HR	95% CI	*P*	HR	95% CI	*p*
All-cause mortality									
CRP	4.16	2.16–8.01	<0.001	2.82	1.72–4.64	<0.001	2.66	1.56–4.49	0.003
Albumin	0.33	0.17–0.66	0.002						
Age	1.06	1.02–1.103	0.008	1.06	1.01–1.11	0.023	1.06	1.01–1.11	0.021
Diabetes	1.19	0.54–2.62	0.667						
IHD	2.16	0.99–4.74	0.054						
SFACS	1.29	0.90–1.86	0.164						
BKACS	1.61	1.05–2.49	0.03						
CLTI	5.06	2.00–12.79	<0.001						
Cardiovascular mortality									
CRP	3.09	1.34–7.137	0.008						
Albumin	0.45	0.17–1.24	0.122						
Age	1.05	0.99–1.11	0.078						
Diabetes	2.72	0.91–8.13	0.076						
IHD	7.25	2.02–26.01	0.002	5.48	1.30–23.06	0.021	5.59	1.32–23.63	0.019
SFACS	1.83	0.98–3.40	0.057						
BKACS	2.28	1.19–4.36	0.013						
CLTI	9.11	3.01–27.56	<0.001						
Cardiovascular event									
CRP	5.36	2.64–10.90	<0.001	2.13	1.37–3.34	<0.001	1.59	1.07–2.37	0.022
Albumin	0.42	0.19–0.92	0.03						
Age	1.06	1.01–1.11	0.014	1.06	1.01–1.12	0.026	1.06	1.00–1.11	0.034
Diabetes	20.1	0.84–4.74	0.117						
IHD	2.15	0.91–5.06	0.803						
SFACS	2.03	1.18–3.49	0.01	1.72	1.05–2.81	0.031			
BKACS	2.29	1.35–3.90	0.002						
CLTI	9.27	3.74–22.94	<0.001						
Limb amputation									
CRP	3.44	1.09–10.87	0.035						
Albumin	0.34	0.09–1.24	0.103						
Age	1.02	0.96–1.09	0.522						
Diabetes	3.79	0.73–19.54	0.112						
IHD	2.61	0.58–11.62	0.211						
SFACS	3.76	1.00–14.19	0.05						
BKACS	4.14	1.30–13.14	0.016						
CLTI	18.61	3.96–87.32	<0.001	12.98	1.63–103.39	0.016	13.94	2.06–94.39	0.007

Abbreviations: CRP, C-reactive protein; IHD, ischemic heart disease; SFACS, superficial femoral artery calcification score; BKACS, below-knee artery calcification score; CLTI, critical limb-threatening ischemia.

**Table 4:** Cox proportional hazard analysis for association with 10-year clinical outcomes.

	Univariate			Multivariate					
					Model 1			Model 2	
	HR	95% CI	*p*	HR	95% CI	*p*	HR	95% CI	*p*
All-cause mortality									
CRP	2.99	1.88–4.76	<0.001	2.24	1.46–3.44	<0.001	2.26	1.48–3.46	<0.001
Albumin	0.49	0.27–0.91	0.023						
Age	1.05	1.03–1.08	<0.001	1.06	1.02–1.08	<0.001	1.06	1.03–1.09	<0.001
Diabetes	1.98	1.18–3.32	0.01						
IHD	1.76	1.05–2.97	0.033						
SFACS	1.28	1.02–1.61	0.034						
BKACS	1.35	1.03–1.78	0.033						
CLTI	5.92	2.74–12.75	<0.001						
Cardiovascular mortality									
CRP	3.26	1.82–5.84	<0.001	1.78	1.09–2.92	0.023	1.66	1.04–2.66	0.033
Albumin	0.87	0.37–2.04	0.747						
Age	1.04	1.01–1.07	0.02				1.04	1.00–1.07	0.041
Diabetes	3.35	1.68–6.68	<0.001						
IHD	3.21	1.67–6.19	<0.001				2.44	1.06–5.58	0.035
SFACS	1.73	1.22–2.46	0.002						
BKACS	1.71	1.20–2.45	0.003						
CLTI	12.96	5.43–30.91	<0.001	4.59	1.10–19.12	0.036	7.06	1.72–28.99	0.007
Cardiovascular event									
CRP	2.68	1.65–4.36	<0.001	1.74	1.19–2.54	0.004	1.56	1.10–2.21	0.012
Albumin	0.94	0.46–1.91	0.867						
Age	1.05	1.02–1.08	0.001	1.04	1.01–1.07	0.014	1.04	1.01–1.08	0.005
Diabetes	2.01	1.14–3.52	0.016						
IHD	1.91	1.08–3.37	0.025						
SFACS	1.54	1.17–2.01	0.002	1.42	1.01–2.17	0.045			
BKACS	1.59	1.18–2.14	0.003						
CLTI	11.22	5.00–25.21	<0.001				3.84	1.13–13.11	0.032
Limb amputation									
CRP	4.63	1.75–12.28	0.002						
Albumin	0.55	0.17–1.83	0.332						
Age	1.01	0.96–1.06	0.782						
Diabetes	6.87	1.48–31.83	0.014						
IHD	1.69	0.52–5.55	0.386						
SFACS	11.21	2.42–51.87	0.002	5.87	1.14–30.18	0.034			
BKACS	4.23	1.76–10.47	0.002						
CLTI	22.95	6.15–85.66	<0.001				7.74	1.32–45.32	0.023

Abbreviations: CRP, C-reactive protein; IHD, ischemic heart disease; SFACS, superficial femoral artery calcification score; BKACS, below-knee artery calcification score; CLTI, critical limb-threatening ischemia.

## Data Availability

The data presented in this study are available on reasonable request. The data are not publicly available because they are the property of Shonan Kamakura General Hospital.
